# Antibacterial effect of nanocurcumin inside the implant fixture: An in vitro study

**DOI:** 10.1002/cre2.348

**Published:** 2020-11-19

**Authors:** Ramin Negahdari, Mohammad Ali Ghavimi, Ali Barzegar, Mohammad Yousef Memar, Ladan Balazadeh, Sepideh Bohlouli, Simin Sharifi, Solmaz Maleki Dizaj

**Affiliations:** ^1^ Department of Prosthodontics, Faculty of Dentistry Tabriz University of Medical Sciences Tabriz Iran; ^2^ Department of Oral and Maxillofacial Surgery, Faculty of Dentistry Tabriz University of Medical Sciences Tabriz Iran; ^3^ Infectious and Tropical Diseases Research Center Tabriz University of Medical Sciences Tabriz Iran; ^4^ Department of Oral Medicine, Faculty of Dentistry Tabriz University of Medical Sciences Tabriz Iran; ^5^ Dental and Periodontal Research Center Tabriz University of Medical Sciences Tabriz Iran

**Keywords:** antibacterial, implant, nanocurcumin, torque

## Abstract

**Objectives:**

Infections after implant placement are the main reasons for the failure of implant treatments. The present study aimed to evaluate the antibacterial effects of nanocurcumin inside the implant fixture against *Escherichia coli*, *Staphylococcus aureus*, and *Enterococcus faecalis*.

**Materials and Methods:**

Twenty seven implants were classified in three groups for testing the antibacterial effect of nanocurcumin, chlorhexidine (as negative control), and distilled water (as negative control). Each group was then divided into three subgroups to study the effect of the applied torque on the antimicrobial effect of nanocurcumin. All implant abutment assemblies were submerged in bacteria suspension and were incubated at 37°C for 24 hours. The contents of each implant were removed to count the colony of bacteria on the surface of plates containing nutrient agar.

**Results:**

Results indicated that the inhibitory rate of bacteria by nanocurcumin was above 99% in all bacteria. Besides, by increasing the amount of applied torque from 10 to 35 N.cm, the CFU of bacteria in exposure to nanocurcumin significantly were decreased (*p*‐value < 0.01).

**Conclusion:**

The results of this study revealed that nanocurcumin can be used inside the implant fixture in order to use antimicrobial effects and further stabilization and success of the implant.

## 1 INTRODUCTION

Peri‐implantitis is the main reason for the failure of the implant treatment plan as a destructive inflammatory process around the osseointegrated implant due to the bacterial colonization. Therefore, microbial leakage is the main cause of peri‐implantitis (Carcuac et al., 2020). Microbial leakage in the abutment implant interface is the main problem in structures of two‐piece implants and is directly related to peri‐implantitis and inflammatory reactions (Lauritano et al., 2020).

Infections after implant placement are the main reasons for the failure of implant treatments. Some recent studies also described that the microbial contamination could happen at the level of implant‐abutment interfaces (IAI) both in implants with healthy and diseased tissue situations (Canullo et al., 2016; Canullo, Peñarrocha‐Oltra, et al., 2015; Cosyn et al., 2011; Tallarico et al., 2017). In 10.3% and 7.3% of patients, the presence of peri‐implantitis was evident from three sites (peri‐implant sulcus, PIS, internal sections of implant linkages, gingival sulcus of the adjacent teeth), together with clinical criteria (hemorrhage during research, depth of probing collar, plaque indicator). In the 53 peri‐implantitis patients, microbial assessment found no significant variations among PIS and PI testing (Canullo et al., 2016). Mechanical and biological complications, such as abutment screw fractions and peri‐implant disorders, are induced by microgap in the IAI (Rismanchian et al., 2012). At different times in IAI of four different Straumann implant abutments, microgap dimensions and microbial permeation have represented a substantial effect on the average microgap size. The microbial incidence for the peri‐implantitis group at three sites was higher and variations in prevalence between various bacteria types were more conspicuous within the connection than in PIS, based on clinical and microbial disparities between stable peri‐implant circumstances and peri‐implantitis (57 patients; 122 implants) (Canullo et al., 2016). In the presence of peri‐implant illnesses, opportunistic pathogens (*Enterococcus faecalis*, *Pseudomonas aeruginosa*) have been reported at the PIS stage with each implant, important variations between the occurrence and quantities of nosocomial bacteria were found throughout the diseases in the gingival sulcus of neighboring teeth and the attachment and abutment on the internal part of each implant (Canullo, Orlato Rossetti, & Penarrocha, 2015). In the management of peri‐implantitis, this can indicate the significance of connection disinfection (Tallarico et al., 2017).

A wide group of microorganisms, from Gram‐positive cocci to Gram‐negative rods, are capable of penetrating and contaminating micro‐gaps (Passos et al., 2013). These microorganisms, which have been identified in various studies as pathogens causing periodontal diseases and peri‐implant infections include *Fusobacterium nucleatum*, *Prevotella intermedia and Peptostreptococcus micros*, *Bacteroides forsythus*, *Actinobacillus actinomycetemcomitans*, *and Porphyromonas gingivalis*, *Escherichia coli*, *Staphylococcus aureus*, *and E. faecalis (*Haghshenas et al., *2015*; Passos et al., *2013*; Van Winkelhoff et al., *2000* *)*.

Curcumin or diferuloylmethane with a chemical formula (1,7‐bis (4‐hydroxy‐3‐mrthoxyphenyl) ‐1,6‐heptadiene‐3,5‐dione) is from the ginger plant family and its rhizome is known as the turmeric (Ammon & Wahl, 1991). It has long been used as a coloring agent in Asia and is used in traditional medicine for many therapeutic purposes (Araujo & Leon, 2001). Various studies have reported antibacterial, antiviral, antifungal, and antimalarial effects of the plant. Due to the extensive antimicrobial effects of this plant and its high safe dose (12 g daily) in clinical trials on humans, it is used as a basis for the production of antimicrobial drugs (Anand et al., 2007; LaColla et al., 1998). For instance, yarns produced by combining curcumin showed antibacterial effects against *S. aureus* by 45% and *E. coli* by 30% in up to 30 times of washing (Han & Yang, 2005). A mixture of curcumin extract with other antibacterial agents has also been used to produce gels and facial emulsions to improve skincare and wound healing (Varaprasad et al., 2011). According to reports, curcumin prevents the growth and proliferation of *E. coli* by inhibiting the creation of FTsZ factor and inhibits SOS responses arising from Levofloxacin in *E. coli* at a concentration of 8 micrograms per milliliter by reducing the expression of βTEM‐1 (Bellio et al., 2014). Furthermore, curcumin can prevent the growth of 10 different strains of methicillin resistant *S. aureus* (MRSA) at concentrations of 125–250 mg per ml. In addition, mixing curcumin with any antibiotic, including ampicillin, oxacillin, and ciprofloxacin, can significantly inhibit the growth of bacteria (Mun et al., 2013). Previous studies also showed that the local uses of curcumin gel decreased gingival inflammation and improved the severity of disease (Farjana et al., 2014; Guimaraes‐Stabili et al., 2019; Malekzadeh et al., 2020). Also, there is evidence that supports curcumin effectively prevents the activation of inflammatory mediators and has therapeutic effects on periodontal diseases (Farjana et al., 2014; Guimaraes‐Stabili et al., 2019).

Despite all beneficial therapeutic effects of curcumin, the bioavailability of this substance is low due to its low solubility in water. It is poorly absorbed orally and can be rapidly metabolized and removed from the circulatory system. There are several ways to resolve these problems; and the use of curcumin nanoparticles is the main way to increase bioavailability for curcumin (Sharifi et al., 2019; A. K. Singh et al., 2013). The field of nanotechnology has been the most active field of research in modern biotechnology and has been significantly developed. It is also used in medicine in the diagnosis and treatment (Yazdani et al., 2018). The main way to increase bioavailability for curcumin is use from nanoparticulated form of curcumin (Sharifi et al., 2020; A. K. Singh et al., 2013).

The present study aimed to evaluate the antibacterial effects of nanocurcumin (curcumin nanocrystals) inside the implant fixture on *E. coli*, *S. aureus*, and *E. faecalis*.

## 2 MATERIALS AND METHODS

### 2.1 Preparation of the nanocurcumin solution

Curcumin nanocrystals, an orange powder with an average size of 95 nm a spherical morphology, was purchased from ALBORZ Company (Tehran, Iran). Then, 600 mg of this powder was weighted and dissolved in 10 ml of distilled water to prepare a solution with concentration of 60 mg/ml. Then, it was stirred for 30 minute in a little dark bottle at room temperature.

### 2.2 Preparation of bacteria

The bacteria were prepared from Faculty of Medicine, Tabriz University of Medical Sciences, Tabriz, Iran. The bacterial suspensions were prepared by cultivating *E. coli ATCC: 25922*, *S. aureus ATCC: 6538*, *and E. faecalis ATCC: 29212* in brain heart infusion (BHI) broth and incubating it for 24 hours at 37°C. Thereafter, the suspension was diluted to reach a density equivalent to 0.5 McFarland standards.

### 2.3 Implant experiment groups

Twenty seven implants (DIO CO., Busan, South Korea) were mounted and were classified in three groups for testing the nanocurcumin solution (60 mg/ml), chlorhexidine (Sigma‐Aldrich Canada Co.) as positive control and distilled water (as negative control). Each group was then divided into three subgroups for testing the impact of applied torque (10, 20, and 35 N.cm) on the antimicrobial effect of tested compounds. The torque tester was used to close each abutment for the desired torque.

### 2.4 Microbial sampling and detection

Under sterile conditions, the implants were removed from their packaging. Subsequently, they were held with sterile pliers to allow a firm torque action and kept in a vertical position. In the nanocurcumin group, nanocurcumin (60 mg/ml) with a volume of 10 μl was applied to the internal cavity of the implant. For negative control, 10 μl of distilled water was injected into the implant fixation and for negative control, 10 μl of chlorhexidine was used. All implant abutment assemblies were submerged in sterile tubes containing 5 ml of bacteria suspension and were incubated at 37°C for 24 hours. After incubation, caps were separated, and abutments were separated from implants by the hexdriver. The contents of each implant were removed to count the colony of bacteria on the surface of plates containing nutrient agar (Quelab, Canada).

After incubation, 0.01 ml of implant contents was inoculated on the TSA plates. In addition, the diluted suspension (1:10, 1:10^2^, 1:10^3^, 10^4^, and 1:10^5^) were prepared using the 0.01 ml of implants content and inoculated on the surface of TSA plates. Plates were incubated for 24 at 35° and CFU was calculated by following formula:

CFU/ml = Number of colony × Dilution of samples × 10^−1^ (because the dilutions were prepared by 0.01 ml (10^−1^ ml of implants content). Plates were selected for counting on which the bacteria colonies grow separately and did not overlap (Desai et al., n.d.). All steps were repeated three times.

### 2.5 Statistical analysis

The results were reported as mean±  SD and frequency (percentage). Data normality was assessed using the Kolmogorov–Smirnov test. Furthermore, the comparison of mean values of CFU in different torques of different types of solutions was performed separately for each type of bacteria using the Kruskal–Wallis test. SPSS 20 was used to analyze data. *p*‐value of less than 0.05 was considered as the significance level.

## 3 RESULTS

Results of the present study indicated that the CFU amounts for nanocurcumin significantly were decreased (*p*‐value < 0.01) comparing to the CFU amounts for water (as negative control). Besides, by increasing the amount of applied torque from 10 to 35 N.cm, the CFU amounts for nanocurcumin significantly were decreased (*p*‐value < 0.01).

**FIGURE 1:**
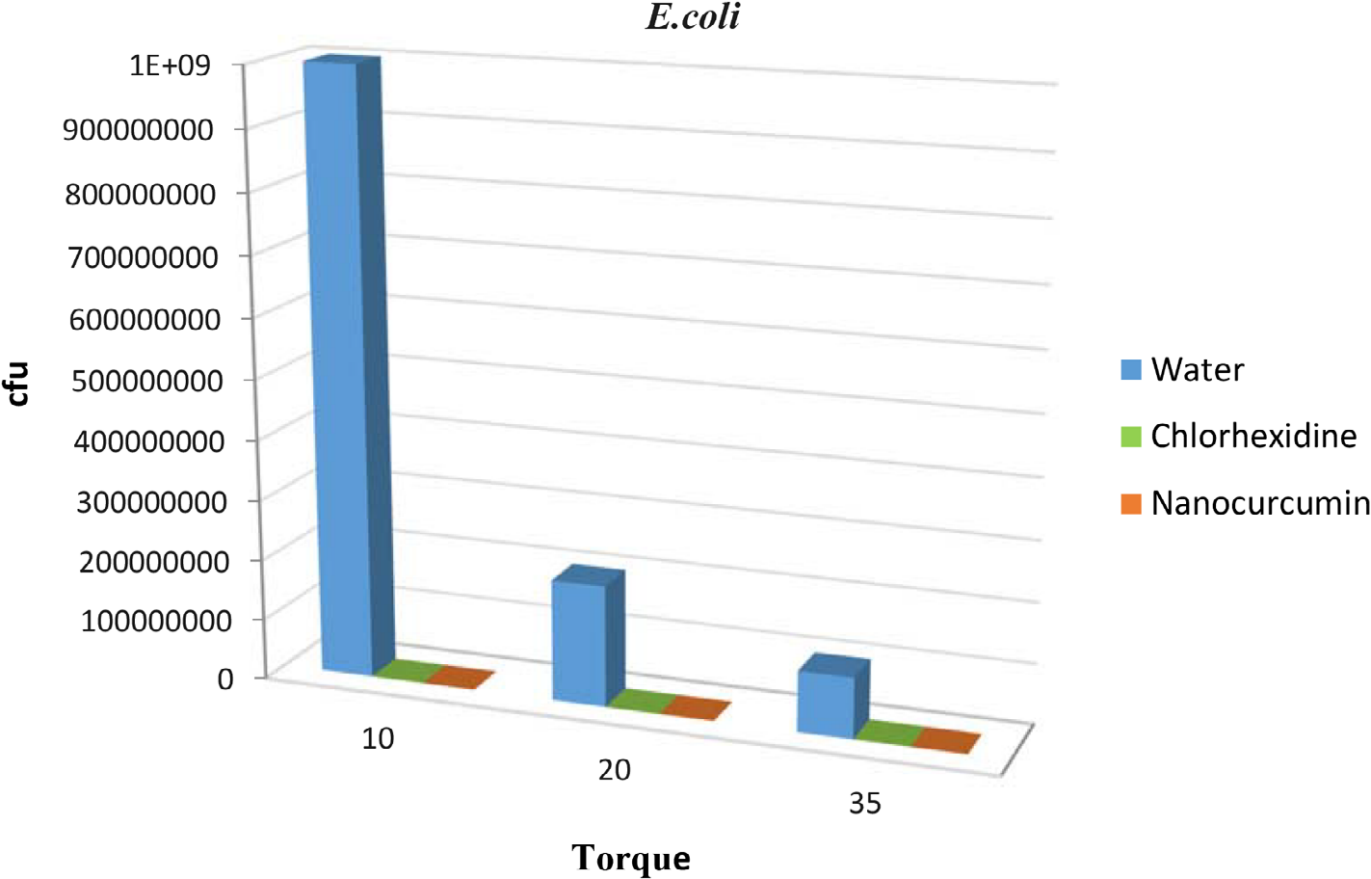
The comparison of the mean growth rates of *Escherichia coli* in different groups

**FIGURE 2:**
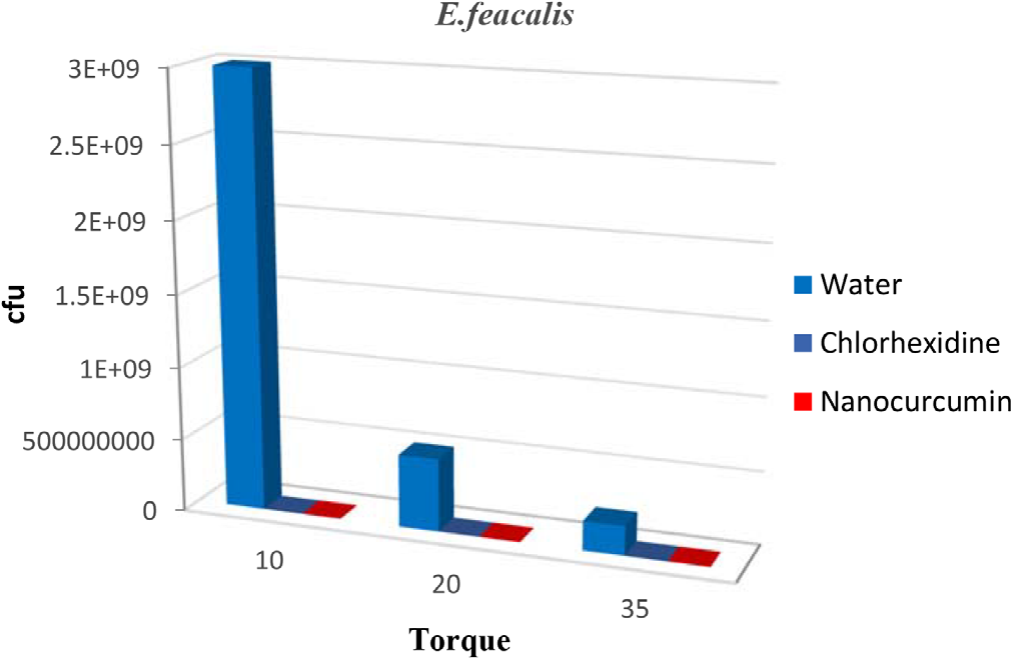
The comparison of the mean growth rates of *Enterococcus faecalis* in different groups

**FIGURE 3:**
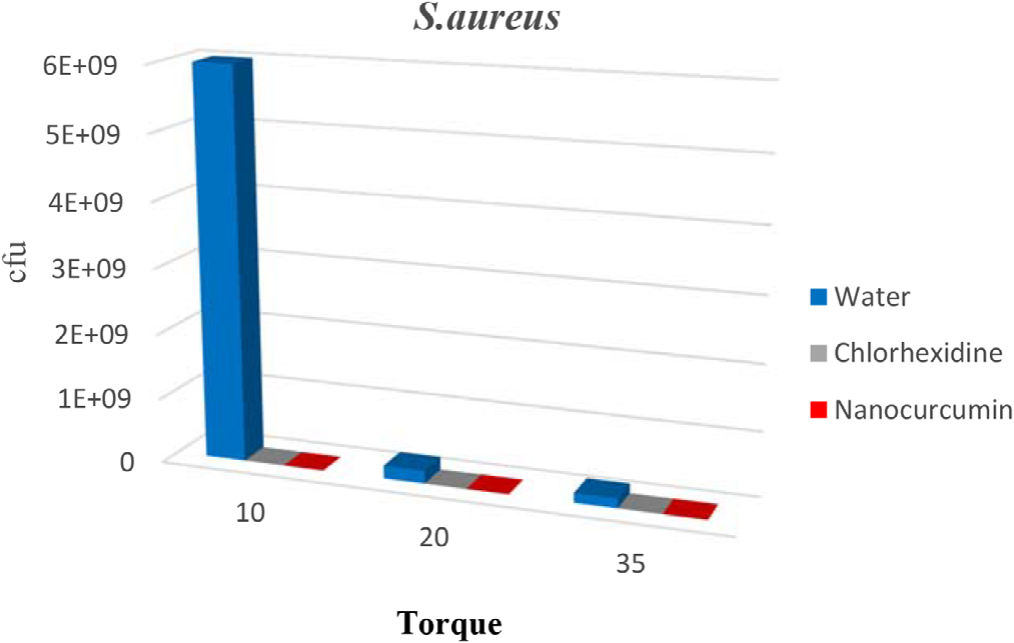
The comparison of the mean growth rates of *Staphylococcus aureus* in different groups

The results also showed that the Inhibitory Rates (IR%) of bacteria by nanocurcumin were higher than 99.99% in all bacteria. Table 1 presents a comparison of inhibitory rates for all three types of bacteria. A comparison of mean growth of bacteria in all three solutions separately for bacteria at different torques is shown in Table 1 and Figures 1, 2, 3.

**TABLE 1:** Comparison of mean growth rates of bacteria in different groups and torques

Bacteria	Torques	Water (CFU)	Chlorhexidine (CFU)	IR *	Curcumin (CFU)	IR**
***Escherichia coli***	10 N.cm	1*10^9^ ± 1.33	30 ± 2.10	99.99	1*10^3^ ± 2.35	99.99
	20 N.cm	2*10^8^ ± 3.11	0	100	2*10^2^ ± 3.75	99.99
	35 N.cm	1*10^8^ ± 0.98	0	100	10 ± 0.89	99.99
***Enterococcus faecalis***	10 N.cm	3*10^9^ ± 1.45	1*10^2^ ± 2.63	99.99	3*10^2^ ± 3.56	99.99
	20 N.cm	5*10^8^ ± 2.83	0	100	0	100
	35 N.cm	2*10^8^ ± 1.01	0	100	0	100
***Staphylococcus aureus***	10 N.cm	6*10^9^ ± 0.59	33.33333	99.99973	5*10^2^ ± 0.99	99.99
	20 N.cm	2*10^8^ ± 1.47	50 ± 3.93	100	2*10^2^ ± 1.01	99.99
	35 N.cm	1.5*10^8^ ± 2.80	140 ± 1.08	100	0	100

*Note*: IR* = (CFU of water – CFU of chlorhexidine)/CFU of water × 100. IR** = (CFU of water – CFU of curcumin)/CFU of water × 100.

## 4 DISCUSSION

A wide group of microorganisms, from gram‐positive cocci to gram‐negative rods, are capable of penetrating and contaminating micro‐gaps (Passos et al., 2013). Recent studies have reported that *S. aureus* can attach the titanium surfaces. This finding justifies the colonization of bacteria on dental implants and the occurrence of secondary infections (Harris & Richards, 2004). In the present study, *E. coli*, *S. aureus*, and *E. faecalis* were used to study the research purpose because of the special properties of these bacteria that were all anaerobic optional bacteria that easily passed through small spaces and micro‐gaps due to their very small sizes and their morphology and also had a very high resistance to harsh conditions such as oxygen and nutrient deficiencies similar to conditions inside the implant fixtures; hence, they were suitable for conditions of our study.

Drug‐carrying nano‐systems have useful capabilities that they did not have before becoming a nanomaterial compound. Nano‐curcumin increases their antimicrobial power against microbes such as *E. coli*, *S. aureus*, *Bacillus subtilis*, *Aspergillus*, and *Saccharomyces cerevisiae*. Curcumin has been also used as an antibiotic against *Yersinia enterocolitica* and *Bacillus cereus* (Bhawana et al., 2011; Y. Wang et al., 2009). Therefore, curcumin nanoparticles can be effective for a longer period of time than providing antibacterial conditions around the implant, and on the other hand, the possible effects of its toxicity will be less due to its safe nature (Bhawana et al., 2011).

The results of the present study indicated that the inhibitory rate of bacteria by nano‐curcumin solution was above 99% in all bacteria. The inhibitory rate of nano‐curcumin solution at all torques showed similar and in some cases better results to chlorhexidine (known as a strong antibacterial agent). Depending on the particle size as well as the type of bacteria, nanoparticles exert their antibacterial effects on bacteria by various mechanisms (Marambio‐Jones & Hoek, 2010). The mechanism is different from what has been reported for the mechanism of action of common antibiotics. To this end, the use of nanoparticles with antimicrobial properties is a way to overcome the microbial resistance of antibiotics. The nanoparticles used in the present study had an average particle size of 95 nm and spherical shape. According to scientific texts, physico‐chemical properties (size, shape and surface properties) and doses of nanoparticles are effective in their antimicrobial effects (Sharifi et al., 2019; Salatin et al., 2015). Studies indicate that nanoparticles in the range below 100 nm generally can disrupt cell membrane functions by binding to the surface of cell membranes with a high affinity compared to larger nanoparticles. Such an effect is more prevalent in smaller nanoparticles due to its larger surface area (Sharifi et al., 2019; L. Wang et al., 2017). The interaction of membrane‐nanoparticles causes local pores in the membrane and largely damages the bacteria due to the entry of nanoparticles into the bacteria and the interaction of intra‐cellular proteins (especially protein rich in sulfur) and DNA. Another possible mechanism for antibacterial effects of antimicrobial nanoparticles is their binding to the bacterial membrane, and their gradual entry into the cytoplasm and disrupting the bacterial functions (Sharifi et al., 2019). Some drug nano‐carriers are also able to mix with the bacterial cell wall and inject their antimicrobial substance into the bacterium (Samiei et al., 2016). Results of a study by Jahromi et al. (2015) indicated that curcumin loading in nanoparticles significantly inhibited *S. aureus* in infectious rat skin. The research results indicated that the use of nanoparticles increased the bactericidal effect of curcumin (Jahromi et al., 2015). In fact, the gradual release of curcumin from nanoparticles occurred at the site of infection and fully inhibited the bacterial infection (Anitha et al., 2011). Results of a study by Bhawana et al. also indicated that curcumin nanoparticles had more antimicrobial effects on a variety of fungi. Moreover, a study by Singh produced similar results and indicated that the antimicrobial and antibacterial properties of curcumin were proven following its use in infectious therapies (R. K. Singh et al., 2010). In another study by Rai et al., who investigated the antibacterial effects of curcumin, the findings indicated that curcumin inhibited the growth of *S. aureus* (Nazemi, Mirzaei, & Jafari, 2019).

Furthermore, results of a study by Desai et al. (2018) on the reduction of bacterial contamination of internal and external dental implants using silver nanoparticles indicated that no bacterial growth was observed in groups containing silver nanoparticles. The absence of bacterial growth in the experimental groups suggested that silver particles were highly effective in inhibiting the bacterial growth inside implants after 24 hours of contact with bacterial suspensions (*E. coli*, *S. aureus*, *and Salmonella*) (Desai et al., n.d.). Our study was very similar to the above study, except that we used a different type of antimicrobial material due to the lack of a study on nano‐curcumin particles and problems caused by implant treatment among different torques, and we thus investigated the simultaneous effects of torques on amount of microbial leakage.

Bacterial colonization in the IAI depends on three factors: type of implant system, dynamic loading, and prophylactic treatment in the interface using disinfectants and flooding agents (Podhorsky et al., 2016). In a study by Podhorsky et al. (2016) on disinfection of the IAI using two types of sealants (Berutemp 500 T2 and Kiero seal) and two types of disinfectant (CHX 1% Gel and GlaxoSmithKline). It was found that the substances could reduce bacterial colonization in the interface, but cannot completely kill bacteria (Podhorsky et al., 2016).

Results of the present study indicated that the CFU amounts for nanocurcumin solution significantly were decreased (*p*‐value < 0.01) comparing to the CFU amounts for water (as negative control). Besides, by increasing the amount of applied torque from 10 to 35 Nm, the CFU amounts for nanocurcumin solution significantly were decreased (*p*‐value < 0.01). Indeed, an increase in torque causes a better seal in the chamber and prevents bacterial entrance. Results of a study by D'Ercole et al., who evaluated the association of bacterial leakage in implants with internal Morse cone connections and the use different amounts of torque in vitro, indicated that an increase in the amount of torque applied to 40 Newtons was directly related to zero microbial leakage (D'Ercole et al., 2014). Our study provided similar results to the above study, while Joao Silva found that different amounts of torque did not have any significant effect on the reduction of microleakage (da Silva‐Neto et al., 2012).

There were some limitations in this study during the experimental steps. For example; the possibility of microbial contamination with other bacteria, the possibility of nanoparticle aggregation, human error in closing the abutment for the desired torque, and the human error in bacteria sampling from the contents of each implant.

## 5 CONCLUSION

Results indicated that the inhibitory rate of bacteria by nanocurcumin was above 99% in all bacteria. Besides, an increase in torque causes a better seal in the chamber and prevents bacterial entrance. However, the present study was an in vitro test and for clinical situations, we need to choose the proper torque according to the different factors. We should provide the correlation between the recommended torque, the situation of the patient, and the prevention of the contamination for clinical investigations. In addition, according to the obtained results of the present study, chemicals can be gradually replaced with antibacterial plant substances such as curcumin in the future. This replacement will not only overcome the microbial resistance but will be also a solution to reduce the use of chemicals and their side effects and toxicity. Then, due to the antimicrobial effects of curcumin as well as its beneficial effects for gingival, nanocurcumin can be used inside the implant fixture to use antimicrobial effects and further stabilization and success of the implant.

## CONFLICT OF INTEREST

The authors declare that they have no conflicts of interests.

## AUTHOR CONTRIBUTIONS

Ramin Negahdari, Mohammad A. Ghavimi, Mohammad Y. Memar, Ali Barzegar, Sepideh Bohlouli and Ladan Balazadeh have made a contribution to the research steps or the drafting of the manuscript. Solmaz Maleki Dizaj and Simin Sharifi had contribution to the drafting and scientifically revision of the manuscript. Solmaz Maleki Dizaj and Simin Sharifi are the corresponding authors of the manuscript. All authors read and approved the final manuscript.

## Data Availability

The raw/processed data required to reproduce these findings cannot be shared at this time as the data also forms part of an ongoing study.
